# Liver Function Changes in Patients with Hepatocellular Carcinoma Treated with Lenvatinib: Predictive Factors of Progression to Child-Pugh Class B, the Formation of Ascites and the Candidates for the Post-Progression Treatment

**DOI:** 10.3390/cancers12102906

**Published:** 2020-10-10

**Authors:** Takeshi Hatanaka, Satoru Kakizaki, Tamon Nagashima, Masashi Namikawa, Takashi Ueno, Hiroki Tojima, Daichi Takizawa, Atsushi Naganuma, Hirotaka Arai, Ken Sato, Norifumi Harimoto, Ken Shirabe, Toshio Uraoka

**Affiliations:** 1 Department of Gastroenterology, Gunma Saiseikai Maebashi Hospital, 564-1 Kamishindenmachi, Maebashi, Gunma 371-0821, Japan; 2 Department of Gastroenterology and Hepatology, Gunma University Graduate School of Medicine, 3-39-15 Showa, Maebashi, Gunma 371-8511, Japan; kakizaki@gunma-u.ac.jp (S.K.); m08702015@gunma-u.ac.jp (H.T.); satoken@gunma-u.ac.jp (K.S.); uraoka@gunma-u.ac.jp (T.U.); 3 Department of Clinical Research, National Hospital Organization Takasaki General Medical Center, 36 Takamatsu-cho, Takasaki, Gunma 370-0829, Japan; 4 Department of Gastroenterology, National Hospital Organization Shibukawa Medical Center, 383 Shirai, Shibukawa, Gunma 377-0280, Japan; nagashima.tamon.tn@mail.hosp.go.jp; 5 Department of Internal Medicine, Kiryu Kosei General Hospital, 6-6-3 Orihime-cho, Kiryu, Gunma 376-0024, Japan; namikawa0501@yahoo.co.jp; 6 Department of Internal Medicine, Isesaki Municipal Hospital, 12-1 Tsunatorihonmachi, Isesaki, Gunma 372-0817, Japan; voluntadycamino@ae.auone-net.jp; 7 Department of Gastroenterology, Maebashi Red Cross Hospital, 389-1 Asakuramachi, Maebashi, Gunma 371-0811, Japan; daichi.takizawa@maebashi.jrc.or.jp (D.T.); h-arai@maebashi.jrc.or.jp (H.A.); 8 Department of Gastroenterology, National Hospital Organization Takasaki General Medical Center, 36 Takamatsu-cho, Takasaki, Gunma 370-0829, Japan; naganuma.atsushi.nj@mail.hosp.go.jp; 9 Department of Hepatobiliary and Pancreatic Surgery, Gunma University Graduate School of Medicine, 3-39-15 Showa, Maebashi, Gunma 371-8511, Japan; nharimotoh1@gmail.com (N.H.); kshirabe@gunma-u.ac.jp (K.S.)

**Keywords:** hepatocellular carcinoma, liver function, albumin-bilirubin grade, lenvatinib, ramucirumab

## Abstract

**Simple Summary:**

Many drugs has become available for the advanced hepatocellular carcinoma and sequential systemic therapies play an important role in the clinical settings. Because the key eligibility criteria for post-progression treatment included the performance status of ≦1 and Child-Pugh score of ≦6, to clarify the eligibility for post-progression treatment will provide meaningful information. The aim of this multicenter retrospective study was to investigate the predictive factors of progression to Child-Pugh class B, the formation of ascites and the candidates for post-progression treatment. Authors confirmed that male, ALBI grade 1, Child-Pugh score 5 (CP5A) and BCLC early or intermediate stage were favorable factors related to sustaining liver function and the patients with ALBI grade 1 and CP5A were eligible for the post-progression treatment. Careful screening for ascites was needed in patients with low platelet count and Child-Pugh score 6.

**Abstract:**

The aim of this multicenter retrospective study was to assess the change in liver function in patients with hepatocellular carcinoma treated with lenvatinib. Among 139 consecutive patients receiving lenvatinib treatment between March 2018 and July 2019, 28 patients with Child-Pugh class B and one patient with inadequate patient information were excluded. Remaining 110 patients with Child-Pugh class A were analyzed. The median age of 110 patients was 73 years (IQR 66.7–80) and 88 patients (80.0%) were men. Child-Pugh score was 5 (CP5A) and 6 (CP6A) in 58 (52.7%) and 52 patients (47.3%), and ALBI grade was 1 and 2 in 38 (34.5%) and 72 patients (65.5%), respectively. The deterioration to Child-Pugh class B was found in 43 patients (39.1%) during the lenvatinib treatment. The favorable factors related to preserving liver function were significantly shown to be male, ALBI grade 1, CP5A and BCLC early or intermediate stage in the multivariate analysis. The formation of ascites was found in 32 patients (28.6%). The significant unfavorable factors associated with the formation of ascites were found to be low platelet count and CP6A. Among the 79 patients, there were 36 (45.6%) and 11 patients (13.9%) who fulfilled the criteria for candidate for the post-progression treatment and ramucirumab treatment, respectively. The predictive factors of the post-progression treatment were shown to be ALBI grade 1 and CP5A in multivariate analysis. In conclusion, male, ALBI grade 1, CP5A and BCLC early or intermediate stage were favorable factors related to sustaining liver function and the patients with ALBI grade 1 and CP5A were eligible for the post-progression treatment. Careful screening for ascites was needed in patients with low platelet count and CP6A.

## 1. Introduction

Liver cancer ranks second as the cause of cancer-related death for men and the sixth as the cause of cancer-related death for women worldwide [1] and hepatocellular carcinoma (HCC) is the most frequent type of primary liver cancer [2]. Sorafenib, an oral multikinase inhibitor, was the first systemic agent to show the survival benefit for patients with advanced HCC [3,4] and has been widely used in clinical settings [5,6,7]. The REFLECT trial [8] revealed that lenvatinib showed non-inferiority to sorafenib in terms of overall survival (OS) and a statistically significant improvement in the objective response rate (ORR) and progression-free survival (PFS). Lenvatinib has recently become available as the first-line systemic therapy and showed the ORR of 30.4 to 39.0 % and the median PFS of 5.4 to 7.6 months in clinical settings [9,10,11,12]. Moreover, regorafenib [13] and cabozantinib [14] demonstrated efficacy in second-line treatment and ramucirumab [15] also provide the better outcome in patients with α-fetoprotein (AFP)≧ 400 ng/mL. These drugs recently have become widely used and sequential systemic therapies play an important role in the clinical setting. Although key eligibility criteria for these second-line treatment included the performance status (PS) of ≦1 and Child-Pugh score of ≦6, there are few previous reports concerning about the frequency and the predictive factors of preserving PS and liver function in HCC patients after receiving lenvatinib. Although the efficacy and safety of post-progression treatment after lenvatinib had not been established yet, to clarify the preserved liver function and the eligibility for post-progression treatment in the clinical settings will provide meaningful information for many physicians. The aim of the current study is to investigate the predictive factors of progression to Child-Pugh class B (CP-B), the formation of ascites and the candidates for post-progression treatment.

## 2. Results

### 2.1. Patient Characteristics

The median age of 110 patients included in this study was 73 years (interquartile range [IQR] 66.7–80) and 88 patients (80.0%) were men. Performance status was 0, 1 and 2 in 77 (70.0%), 27 (24.5%) and 6 patients (5.5%), respectively. About half of patients had HCV-associated liver disease. Among 12 patients infected with HBV, eight patients (66.7%) received antiviral therapy and HCV eradication was achieved in 32 patients (55.2%). Child-Pugh score was 5 (CP5A) and 6 (CP6A) in 58 (52.7%) and 52 patients (47.3%), and the albumin-bilirubin (ALBI) grade was 1 and 2 in 38 (34.5%) and 72 (65.5%), respectively. 20 patients (18.8%) were treatment-naïve and 93 patients (84.5%) was received transcatheter arterial chemoembolization (TACE) procedure previously. Esophageal varices were none, F1 and F2 in 82 (76.6%), 16 (15.0%) and nine patients (8.4%), respectively. Three patients (2.7%) were not received esophagogastroduodenoscopy. Nine patients (8.2%) had the history of treatment for esophageal varices. The initial full dose and reduced dose of lenvatinib was in 58 (52.7%) and 52 patients (47.3%), respectively. Regarding tumor factors, BCLC stage was early, intermediate and advanced stage in 3 (2.7%), 33 (30.0%) and 74 patients (67.3%), respectively. There were 36 patients (32.7%) with extrahepatic spread and 22 patients (20.0%) with macroscopic vascular invasion (MVI). There were no patients with presence or the history of ascites. Patient characteristics are summarized in Table 1.

### 2.2. Tumor Response, PFS and OS

**Figure 1:**
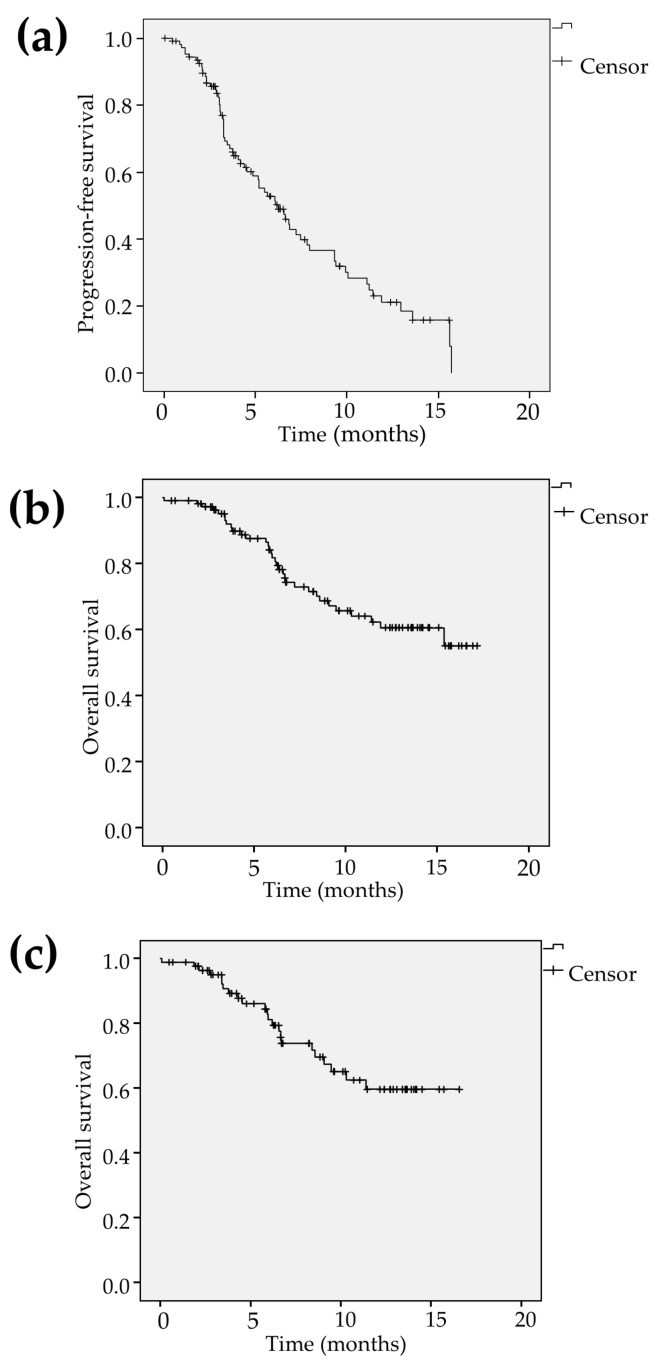
(**a**) Kaplan-Meier curve showed that the median progression-free survival (PFS) in all patients was estimated to be 6.3 months (95% confidence interval [CI] 4.7–7.8) and the PFS event were found in 68 patients (61.8%) at the time of analysis. (**b**) The 6- and 12-month overall survival (OS) rates were calculated to be 81.8% (95% CI 73.8–89.8) and 60.6% (95% CI 49.4–71.8) in all patients. The median OS was not reached during the observation. The OS events were observed in 33 patients (30.0%). (**c**) Among the patients treated with lenvatinib as the first-line treatment (*n* = 84), the 6- and 12-month survival rates were shown to be 81.1% (95% CI 71.7–90.5) and 59.7% (95% CI 46.2–73.2) and the OS events were seen in 23 patients (27.4%).

Tumor response assessed by the modified Response Evaluation Criteria in Solid Tumors (mRECIST) was determined as complete response (CR), partial response (PR), stable disease (SD) and progressive disease (PD) in 1 (1.1%), 26 (28.9%), 44 (48.9%) and 19 patients (21.1%), respectively. Therefore, ORR and disease control rate (DCR) were calculated to be 30.0%, 83.3%, respectively (Table 2). The median follow-up time in all patients was estimated to be 7.0 months (4.1–12.9). The median PFS was calculated to be 6.3 months (95% confidence interval [CI] 4.7–7.8) and the event were found in 68 patients (61.8%) at the time of analysis (Figure 1a). The 6- and 12-month survival rates were calculated to be 81.8% (95% CI 73.8–89.8) and 60.6% (95% CI 49.4–71.8) in all patients. The median OS was not reached during the observation. The OS events were observed in 33 patients (30.0%; Figure 1b). Among the patients treated with lenvatinib as the first-line treatment (*n* = 84), the 6- and 12-month survival rates were shown to be 81.1% (95% CI 71.7–90.5) and 59.7% (95% CI 46.2–73.2) and the OS events were seen in 23 patients (27.4%; Figure 1c). The significant predictive factor relevant to OS was found to be BCLC advanced stage (hazard ratio [HR] 2.50, 95% CI 1.02–6.10, *p* = 0.045 in model 3) in multivariate analysis while there were no significant factors in patients treated with lenvatinib as the first-line treatment (Table 3).

### 2.3. Changes in Parameters Associated with Liver Function

**Figure 2:**
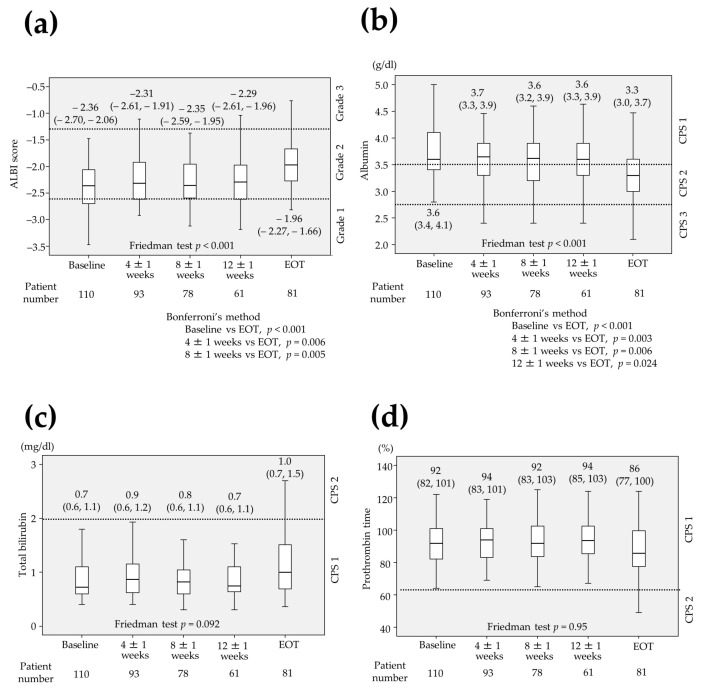
Changes in parameters associated with liver function. (**a**) albumin-bilirubin (ALBI) score (**b**) serum albumin (**c**) total bilirubin (**d**) prothrombin time. (**a**,**b**) The ALBI score and the serum albumin were significantly differed in 42 patients in whom all laboratory data were measured at baseline (*p* < 0.001, *p* < 0.001, respectively). The significant differences were observed between the ALBI at baseline and at end of treatment (EOT) (*p* < 0.001), that at 4 ± 1 weeks and at EOT (*p* = 0.006), that at 8 ± 1 weeks and at EOT (*p* = 0.005). There were also significant differences between the serum albumin at baseline and EOT (*p* < 0.001), that at 4 ± 1 weeks and at EOT (*p* = 0.003), that at 8 ± 1 weeks and at EOT (*p* = 0.006), that at 12 ± 1 weeks and at EOT (*p* = 0.024). (**c**,**d**) Total bilirubin and prothrombin time were not significantly differed (*p* = 0.092, *p* = 0.95, respectively). CPS: Child-Pugh score.

The median ALBI score was calculated to be −2.36 (range −2.70 to −2.06) at baseline (*n* = 110), −2.31 (−2.61 to −1.91) at 4 ± 1 weeks (*n* = 93), −2.35 (−2.59 to −1.95) at 8 ± 1 weeks (*n* = 78), −2.29 (−2.61 to −1.96) at 12 ± 1 week (*n* = 61) and −1.96 (−2.27 to −1.66) at end of treatment (EOT) (*n* = 81), respectively (Figure 2a). The albumin was 3.6 g/dL (3.4–4.1), 3.7 g/dL (3.3–3.9), 3.6 g/dL (3.2–3.9), 3.6 g/dL (3.3–3.9) and 3.3 g/dL (3.0–3.7) at baseline, 4 ± 1 weeks, 8 ± 1 weeks, 12 ± 1 weeks and EOT, respectively (Figure 2b) while total bilirubin was 0.7 mg/dL (0.6–1.1), 0.9 mg/dL (0.6–1.2), 0.8 mg/dL (0.6–1.1), 0.7 mg/dL (0.6–1.1) and 1.0 mg/dL (0.7–1.5) (Figure 2c) and prothrombin time was 92% (82–101), 94% (83–101), 92% (83–103), 94% (85–103) and 86% (77–100) at baseline, 4 ± 1 weeks, 8 ± 1 weeks, 12 ± 1 weeks and EOT, respectively (Figure 2d). Among 42 patients whose laboratory data were measured at baseline, 4 ± 1 weeks, 8 ± 1 weeks, 12 ± 1 weeks and EOT without missing data, the ALBI score and the albumin were significantly differed (*p* < 0.001, Friedman test) whereas total bilirubin and prothrombin time were not. The significant differences were observed between the ALBI at baseline and at EOT (*p* < 0.001), that at 4 ± 1 weeks and at EOT (*p* = 0.006), that at 8 ± 1 weeks and at EOT (*p* = 0.005, Bonferroni’s method; Figure 2a). There were also significant differences between the albumin at baseline and EOT (*p* < 0.001), that at 4 ± 1 weeks and at EOT (*p* = 0.003), that at 8 ± 1 weeks and at EOT (*p* = 0.006), that at 12 ± 1 weeks and at EOT (*p* = 0.024, Bonferroni’s method; Figure 2b).

### 2.4. An Analysis of the Deterioration to CP-B

**Figure 3:**
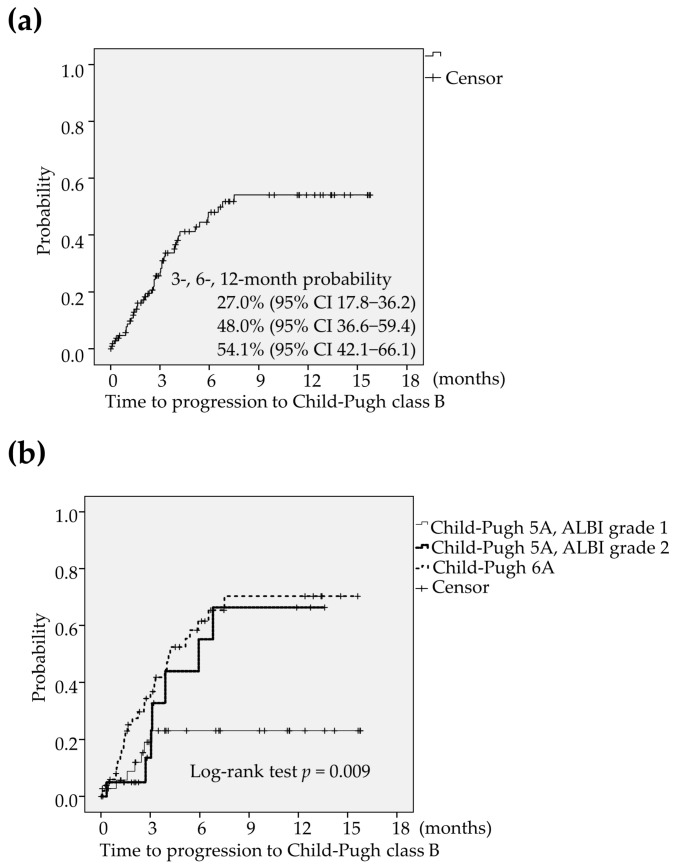
(**a**) The deterioration to CP-B was found in 43 patients (39.1%) during the lenvatinib treatment. The Kaplan-Meier estimates showed that the 3-, 6-, and 12-month probability of time to progression to CP-B was calculated to be 73.0% (95% CI 63.8–82.2), 52.0% (95% CI 40.6–63.4) and 45.9% (95% CI 33.9–57.9), respectively. (**b**) Liver function was preserved better in patients with CP5A-G1 compared to those with CP5A-G2, and those with CP6A (*p* = 0.009). CI: confidence interval, CP-B: Child-Pugh class B, CP5A-GI: Child-Pugh score 5 and ALBI grade 1, CP5A-G2: Child-Pugh score 5 and ALBI grade 2, CP6A: Child-Pugh score 6.

The deterioration to CP-B was found in 43 patients (39.1%) during the lenvatinib treatment. The 3-, 6-, and 12-month probability of time to progression to CP-B was estimated to be 73.0% (95% CI 63.8–82.2), 52.0% (95% CI 40.6–63.4) and 45.9% (95% CI 33.9–57.9), respectively (Figure 3a). The median period of progression to CP-B was calculated to be 6.8 months. The optimum cut-off value of platelet count for predicting the progression to CP-B were determined using receiver operating characteristic (ROC) curve analysis. When the platelet count was 12.1 × 10^4^/μL, Youden Index was maximized. Accordingly, the optimum cut-off value of the platelet count was set at 12 × 10^4^/μL. The multivariate analysis revealed that the favorable factors related to preserving liver function were significantly shown to be male (female; HR 2.27, 95% CI 1.12–4.54, *p* = 0.021 in model 1, HR 2.24, 95% CI 1.11–4.53, *p* = 0.024 in model 2 and HR 2.33, 95% CI 1.17–4.65, *p* = 0.016 in model 3), ALBI grade 1 (ALBI grade 2; HR 2.38, 95% CI 1.09–5.26, *p* = 0.030 in model 1 and HR 2.35, 95% CI 1.07–5.15, *p* = 0.033 in model 3), CP5A (CP 6A; HR 2.03, 95% CI 1.04–3.95, *p* = 0.037 in model 2) and BCLC intermediate or early stage (BCLC advanced stage; HR 2.08, 95% CI 1.01–4.17, *p* = 0.048 in model 1;Table 4). Liver function was preserved better in patients with CP5A and ALBI grade 1 (CP5A-G1) compared to those with CP5A and ALBI grade 2 (CP5A-G2), and those with CP6A (*p* = 0.009; Figure 3b).

### 2.5. An Analysis of the Formation of Ascites

**Figure 4:**
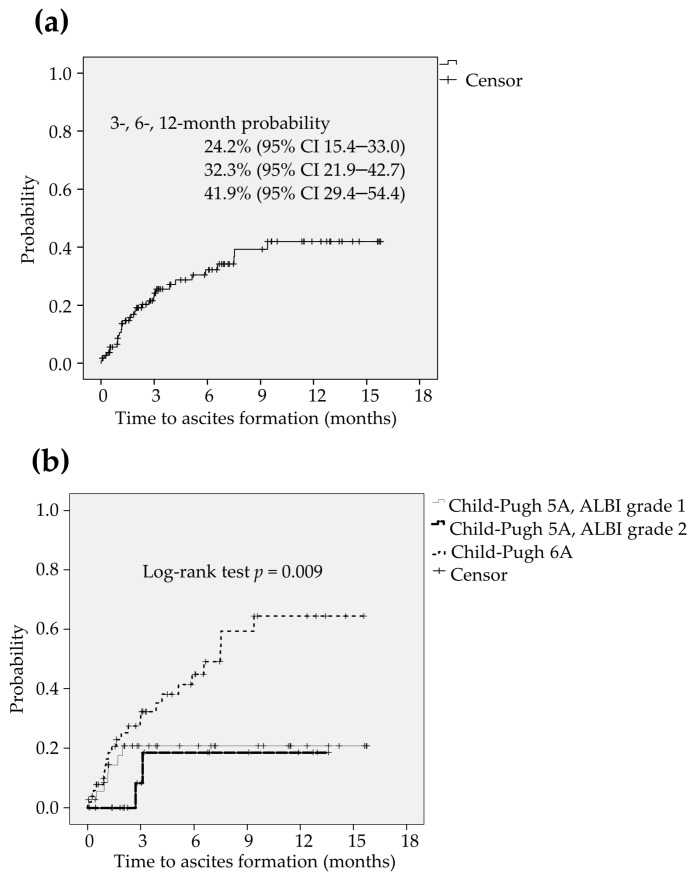
(**a**) The formation of ascites was found in 32 patients (28.6%) during the lenvatinib treatment. The 3-, 6-, and 12-months probability of time to ascites formation was estimated to be 24.2% (95% CI 15.4–33.0), 32.3% (95% CI 21.9–42.7) and 41.9% (95% CI 29.4–54.4), respectively. (**b**) The formation of ascites was more frequently found in patients with CP6A than those with CP5A-G1 and those with CP5A-G2 (*p* = 0.009). CI: confidence interval, CP5A-GI: Child-Pugh score 5 and ALBI grade 1, CP5A-G2: Child-Pugh score 5 and ALBI grade 2, CP6A: Child-Pugh score 6.

The formation of ascites was also found in 32 patients (28.6%) during the lenvatinib treatment. The 3-, 6-, and 12-months probability of time to ascites formation was estimated to be 24.2% (95% CI 15.4–33.0), 32.3% (95% CI 21.9–42.7) and 41.9% (95% CI 29.4–54.4), respectively (Figure 4a). The median period of ascites formation was not reached. The optimum cut-off value of platelet count for predicting the formation of ascites were determined using ROC curve analysis. When the platelet count was 11.8 × 10^4^/μL, Youden Index was maximized. Accordingly, the optimum cut-off value of the platelet count was also set at 12 × 10^4^/μL. The significant unfavorable factors associated with the formation of ascites were found to be low platelet count (HR 2.56, 95% CI 1.22–5.26, *p* = 0.013 in model 1, HR 2.63, 95% CI 1.25–5.26, *p* = 0.010 in model 2) and CP6A (HR 2.17, 95% CI 1.03–4.55, *p* = 0.041 in model 2) in multivariate analysis (Table 5). The formation of ascites was more frequently found in patients with CP6A than those with CP5A-G1 and those with CP5A-G2 (*p* = 0.009; Figure 4b).

### 2.6. Comparsion between the Liver Function in SVR Patients and Non-SVR Patients

**Figure 5:**
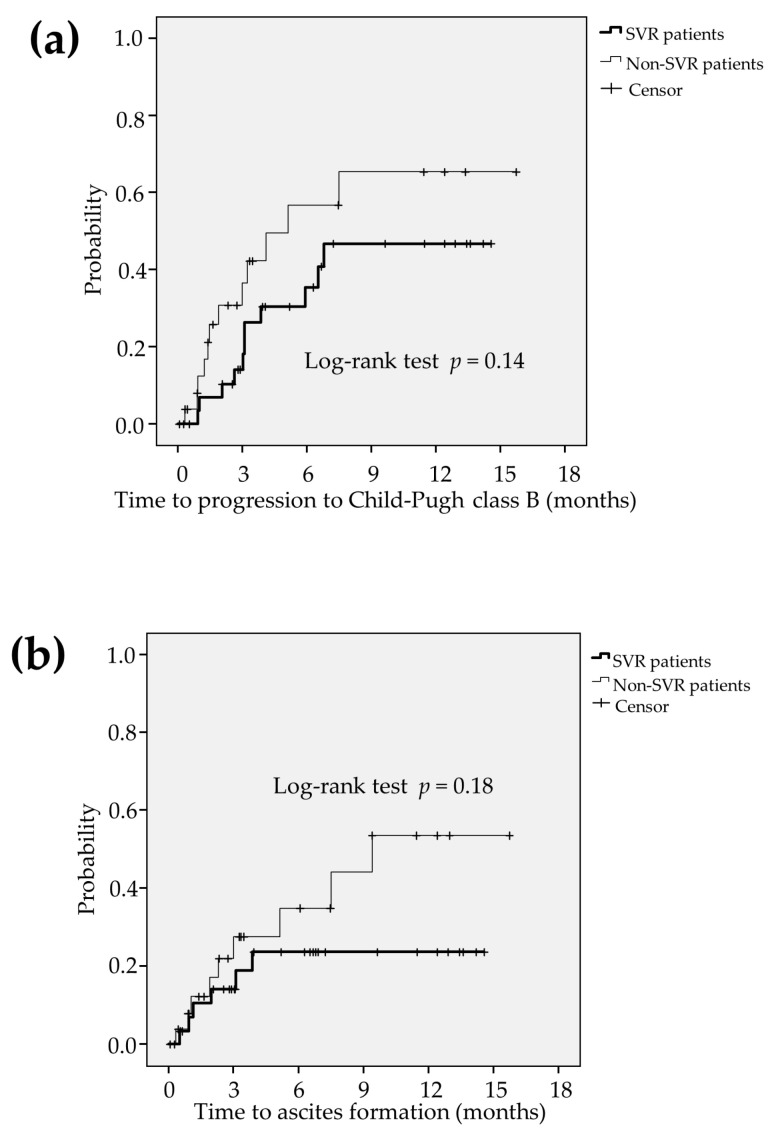
Among HCC patients infected with HCV (*n* = 58), HCV eradication was achieved in 32 patients (55.2%) (defined as SVR patients) and HCV-persistent infection was found in 26 patients (44.8%) (defined as non-SVR patients). During the lenvatinib treatment, there were not significant differences in time to progression to CP-B (*p* = 0.14; (**a**)) and formation of ascites (*p* = 0.18; (**b**)) between the SVR patients and non-SVR patients. CP-B: Child-Pugh class B, HCC: hepatocellular carcinoma, HCV: hepatitis C virus, SVR: sustained virological response.

Among HCV-related HCC patients (n = 58), HCV eradication was achieved in 32 patients (55.2%) (defined as SVR patients) and HCV-persisitent infection was found in 26 patients (44.8%) (defined as non-SVR patients). During the lenvatinib treatment, there were not significant differences in time to progression to CP-B (*p* = 0.14; Figure 5a) and formation of ascites (*p* = 0.18; Figure 5b) between the SVR patients and non-SVR patients.

### 2.7. An Analysis of the Hepatic Decompensation

**Figure 6:**
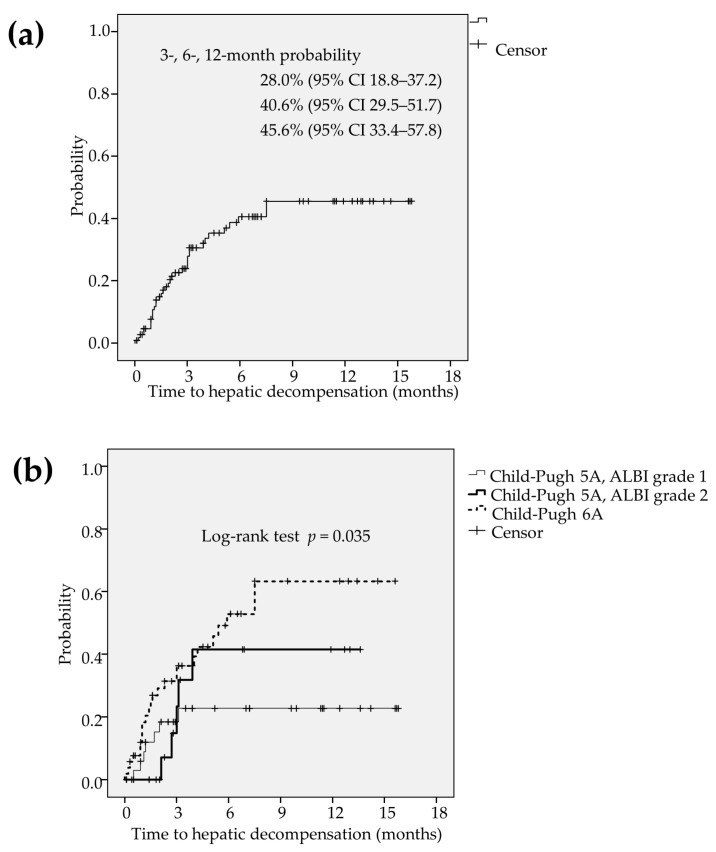
(**a**) The hepatic decompensation was observed in 36 patients (32.7%). Kaplan-Meier curve estimated that the 3-, 6-, and 12-months probability of time to hepatic decompensation were 28.0% (95% CI 18.8–37.2), 40.6% (95% CI 29.5–51.7) and 45.6% (95% CI 33.4–57.8), respectively. (**b**) Hepatic decompensation was less frequently found in patients with CP5A-G1 compared to those with CP5A-G2 and those with CP6A (*p* = 0.035). CI: confidence interval, CP5A-GI: Child-Pugh score 5 and ALBI grade 1, CP5A-G2: Child-Pugh score 5 and ALBI grade 2, CP6A: Child-Pugh score 6.

During the observation, the hepatic decompensation was observed in 36 patients (32.7%). Kaplan-Meier curve estimated that the 3-, 6-, and 12-months probability of time to hepatic decompensation were 28.0% (95% CI 18.8–37.2), 40.6% (95% CI 29.5–51.7) and 45.6% (95% CI 33.4–57.8), respectively (Figure 6a). Hepatic decompensation was less frequently found in patients with CP5A-G1 compared to those with CP5A-G2 and those with CP6A (*p* = 0.035; Figure 6b).

### 2.8. Candidate for the Post-Progression Treatment and Ramucirumab Treatment

**Figure 7:**
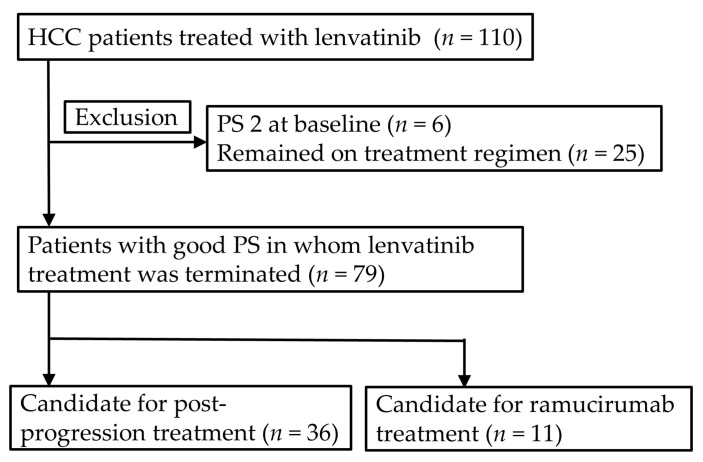
Among 79 patients with PS 0-1 who terminated lenvatinib treatment at the time of analysis, there were 36 patients (45.6%) who fulfilled the criteria for candidate for post-progression treatment such as regorafenib and cabozantinib (patients with preserved PS and liver function) and 11 patients (13.9%) who the criteria for candidate for ramucirumab treatment (patients with preserved PS, liver function and serum level of α-fetoprotein ≧ 400 ng/ml). HCC: hepatocellular carcinoma, PS: performance status.

In the analysis of the candidate for the post-progression treatment and ramucirumab treatment, authors excluded six patients (5.5%) with PS 2 and 25 patients (22.7%) whose lenvatinib treatment was continued at the time of analysis. Therefore, among the remaining 79 patients, there were 36 patients (45.6%) and 11 patients (13.9%) who fulfilled the criteria for candidates for the post-progression treatment and ramucirumab treatment, respectively (Figure 7). Multivariate analysis revealed that the favorable predictive factors of the post-progression treatment were shown to be ALBI grade 1 (odds ratio [OR] 3.24, 95% CI 1.20–8.76, *p* = 0.020 in model 1), CP5A (OR 3.80, 95% CI 1.45–9.95, *p* = 0.007 in model 2) and patients initially received full dose (OR 2.92, 95% CI 1.15–7.44, *p* = 0.024 in model 3; Table 6). The lower median ALBI score, higher percentage of ALBI grade 1 and better PS were more frequently found in patients initially received full dose compared to those received reduced dose (Table 7).

## 3. Discussion

The major findings of this study were that predictive favorable factors of preserving liver function were male, ALBI grade 1, CP5A and BCLC intermediate or early stage, and those of the post-progression treatment were ALBI grade 1 and CP5A. While total bilirubin and prothrombin time were not significantly different, the ALBI score and albumin were significantly decreased during lenvatinib treatment. The predictive factors of the formation of ascites were low platelet count and CP6A. The decreased serum level of albumin and the formation of ascites considerably contributed to the deterioration to CP-B.

There has been little study done concerning changes in liver function during lenvatinib treatment. Hiraoka et al reported that the Child-Pugh class A (CP-A) at baseline deteriorated to the CP-B after 4 weeks in 22.6% patients and the median ALBI score at baseline was significantly increased compared to that at 2 weeks and 4 weeks (−2.36 ± 0.45 to –2.20 ± 0.49 at 2 weeks, –2.15 ± 0.50 at 4 weeks, *p* < 0.001) although the observation period of their study was limited to 4 weeks [16]. The results of the present study corresponded with those of their study concerning the analysis of deterioration of the CP-B, but differed from those regarding the ALBI score. The lack of statistical significance in ALBI score could be presumed to be due to the small number of patients (only 42 patients) in the present study. Moreover, they also reported that liver function was preserved better in patients with ALBI grade 1 than those with mALBI grade 2a or 2b, and mALBI grade 1 + 2a was the only predictive factor in a multivariate analysis [17]. The results of their study were consistent with those of the present study.

The low platelet count and CP6A were significant unfavorable factors relevant to the formation of ascites during the lenvatinib treatment. While the formation of ascites was obviously found in patients with poorer liver function than those with a better one, the association of the low platelet count with the formation of ascites remains unclear. The authors’ speculation is that stellate cells play an important role in the regulation of the intrahepatic blood flow [18] and some vasoregulatory compounds including nitric oxide (NO), prostaglandin and endothelin had an effect on these cells [18,19,20]. A reduction in NO contributed to the increase in intrahepatic blood blow [20,21,22] and VEGF induced NO production by endothelial NO synthase [23]. Accordingly, lenvatinib inhibited the production of NO, leading to decrease to intrahepatic blood flow and increased intrahepatic vascular resistance. Although the pathogenesis of thrombocytopenia was multifactorial in patients with chronic liver disease, one of the possible causes arose from increased pooling of platelets in the enlarged spleen due to the portal hypertension [24]. Administration of lenvatinib was presumed to cause the further increase in the portal hypertension in patients with low platelet count, resulting in the formation of ascites.

There were no previous reports associated with the candidate for the post-progression treatment and ramucirumab treatment after receiving lenvatinib treatment. According to the previous studies concerning the 2nd-line treatment after sorafenib treatment, 35∼59% patients met the criteria for candidate for the 2nd-line treatment [25,26,27,28,29]. These previous studies also demonstrated that the predictive factors of the 2nd-line treatment were shown to be male [29], CP5A [25,29], PS 0 [25], MVI [26], hypoalbuminemia [26,28], AlBI score [27,28], small deterioration of liver function after sorafenib treatment [28]. In other words, good liver function, good PS, less advanced HCC stage and male could play a crucial role in transition to the post-progression treatment after sorafenib. In the current study, authors adapted these factors and performed multivariate analyses relevant to progression to CP-B, formation of ascites and post-progression treatment. In this connection, authors also adapted the previous TACE procedure and initial dose of lenvatinib which may impact on liver function and post-progression treatment.

Child-Pugh score 5, ALBI grade 1 and initial full dose were significantly found to be favorable factors of the post-progression treatment after lenvatinib treatment. While CP5A and ALBI grade 1 was consistent to the previous studies [25,27,28,29], initial full dose seemed to be quite different because patients initially received full dose were more likely to worsen the liver function and PS than those received reduced dose. This is probably because the patients with good liver function and PS were initially received full dose, as shown in Table 7, resulting in well-preserved liver function and PS after lenvatinib treatment. While dose reduction or starting dose was not influence on the decreased efficacy of sorafenib therapy [30], relative dose intensity was associated with efficacy of lenvatinib treatment [31,32] and adverse events were manageable in patients with good liver function [11,12]. Therefore, the patients with good liver function were likely to receive initial full dose of lenvatinib, indicating that the initial full dose was found to be significant factor. Accordingly, authors emphasized that good liver function and PS play an important role in post-progression treatment.

While Kuzuya et al reported that only serum level of AFP ≧ 400 ng/mL was shown to be significant factor associated with the candidate ramucirumab treatment after sorafenib treatment [29], multivariate analysis could not be performed in the current study due to the small number of events. Further studies will be conducted to investigate the predictive factors of ramucirumab treatment after lenvatinib treatment.

Cabibbo et al reported that early hepatic decompensation impact on unfavorable outcome on patients with HCV-related early HCC [33]. In the present study, the hepatic decompensation was observed in HCC patients treated with lenvatinib, especially in those with CP6A. Accordingly, lenvatinib could be good indication for the patients with well-preserved liver function in terms of hepatic decompensation.

The prognostic factors in patients treated with lenvatinib was previously reported to be mALBI 1+2a [10], which was inconsistent to the present study. This difference may be due to the lack of statistical power. The large number of cases and adequate follow-up period was needed to clarify the prognostic factors of patients receiving lenvatinib treatment.

Among HCV-related HCC patients, SVR patients tend to sustain liver function during the lenvatinib treatment although significant difference was not found. Accordingly, direct-acting antiviral agent (DAA) for HCV might influence on the maintaining liver function and DAA treatment after curative treatment for the early stage HCC could play an important role in prolongation of overall survival.

Nowadays, lenvatinib is more commonly used than sorafenib in Japan. This is probably because lenvatinib showed a higher ORR and longer PFS compared to sorafenib according to the REFLECT study [8]. However, the efficacy and safety of post-progression treatment after lenvatinib had not been established yet. In addition, question has remained unanswered which treatment (lenvatinib or sorafenib) as the first-line treatment is the better overall survival. Accordingly, the further study was needed to establish the post-progression treatment after lenvatinib in the clinical settings.

In the present study, three patients at BCLC early stage were included. They did not receive curative treatment for the following reasons: one patient refused to receive surgical treatment due to old age. Radiofrequency ablation and TACE could not be performed due to the adhesion between the tumor and gastrointestinal tract and due to an allergic reaction to contrast medium, respectively. Another patient was judged to unresectable due to comorbidities and the tumor was adjacent to the bile duct, leading to be high risk location for RFA. The remaining patient was at potentially high risk for postoperative hepatic failure and RFA was not carried out because a puncture route was not found on ultrasonography. That is why they did not receive curative treatment.

Ten patients (9.1%) with bile duct invasion were included in the present study. These patients were excluded in The REFLECT trial [8] and were likely to cause the obstructive jaundice and acute cholangitis which may affect the deterioration of liver function. In the present study, bile duct invasion was not found to be significant factor possibly due to the small number of cases. Accordingly, careful interpretation was needed to explore the present results.

The current study has some limitations. It is a retrospective cohort study. In addition, the number of patients was small and the observation time was limited. Accordingly, the length of observation period may impact the results. Further study is required to validate the presented results and clarify the factors affecting liver function.

## 4. Materials and Methods

### 4.1. Patients

**Figure 8:**
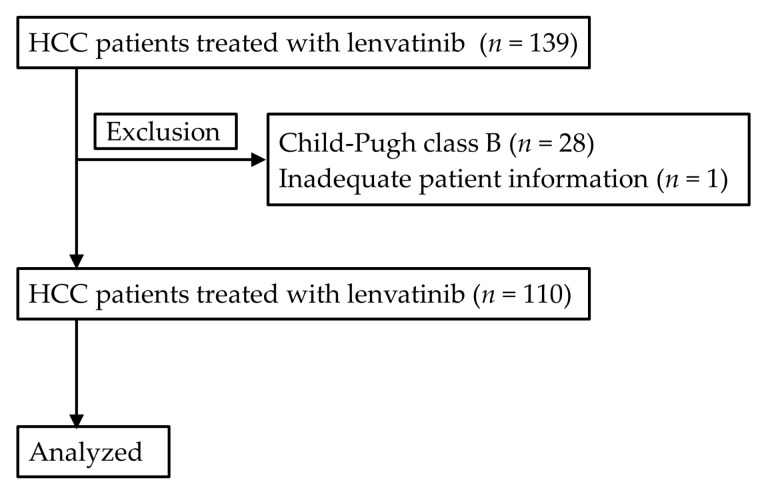
A flowchart of patient selection.

In this retrospective cohort study, HCC patients treated with lenvatinib between March 2018 and July 2019 at Gunma Saiseikai Maebashi Hospital (Maebashi, Gunma, Japan) and its affiliated hospitals were analyzed. Among 139 consecutive patients, 28 patients with CP-B and one patient with inadequate patient information were excluded. Accordingly, remaining 110 patients were included in this study (Figure 8). Diagnosis of HCC was done based on the typical radiological findings according to the criteria of American Association Study of the Liver Diseases [34,35]; that is, enhancement on arterial phase and washout on portovenous or delayed phase on multiphase contrast enhanced imaging such as dynamic computer tomography (CT), gadolinium ethoxybenzyl diethlenetriamine pentaacetic acid (Gd-EOB-DTPA)-enhanced magnetic resonance imaging (MRI). Diagnosis of HCC was also confirmed by pathological findings when tumors showed untypical radiological findings. This study was approved by the institutional review board (2018-11-017) in each institution in accordance with the Declaration of Helsinki, and the need for written informed consent was waived because of the retrospective nature of the study.

### 4.2. Lenvatinib Treatment

All lenvatinib treatment decisions, including the timing of treatment, initial dose, dose reduction during treatment and duration of therapy, were left to the sole discretion of the attending physician. In general, lenvatinib was used more commonly than sorafenib because lenvatinib showed a higher ORR and longer PFS compared to sorafenib according to the phase Ⅲ trial [8]. Lenvatinib was orally administered 12 mg/day (body weight ≧ 60 kg) or 8 mg/day (body weight < 60 kg). Patients were followed up every 2–4 weeks by physical examination, urinalysis and blood examination. Serum level of AFP and des-γ-carboxy prothrombin (DCP) was measured every month and the contrast enhanced imaging such as CT or MRI was performed every 1–3 months. Lenvatinib treatment was terminated upon appearance of tumor progression or unacceptable adverse events.

### 4.3. Assessment of the Liver Function and the Efficacy of Lenvatinib Treatment

The liver function in the patients with HCC was evaluated by Child-Pugh classification and the ALBI grade. The ALBI score calculated using serum level of albumin and total bilirubin at baseline by the following formula: ALBI score = (log_10_ bilirubin [μmol/L] × 0.66) + (albumin [g/L] × –0.085) [36]. The ALBI grades were assigned as follows: ≤–2.60 = grade 1, >–2.60 to –1.39 = grade 2, >–1.39 = grade 3 [36]. Time to progression to CP-B was defined as period from the lenvatinib treatment to deterioration to CP-B. Time to ascites formation was defined as period from the lenvatinib treatment to the appearance of ascites formation. The assessment of liver function was also carried out at the time of lenvatinib discontinuation.

Tumor response was evaluated by mRECIST. Objective response rate was defined as the proportion of patients who achieve the CR and PR, DCR was also defined as that of patients who achieve the CR, the PR and SD. Progression-free survival was defined as period from the lenvatinib treatment to the presence of disease progression or death and OS as period from the lenvatinib treatment to the death or last visit.

### 4.4. Eligibility Criteria for Candidate for Post-Progression Treatment

The criteria for candidate for the post-progression treatment such as regorafenib and cabozantinib was defined as CP-A and PS 0-1 at the time of lenvatinib discontinuation [37]. The criteria for candidate for ramucirumab treatment was also defined as CP-A, PS 0-1 and serum level of AFP ≧ 400 ng/mL according to the eligibility criteria in the REACH-2 trial [15,38].

### 4.5. Statistical Analysis

Continuous variables were presented as the median (IQR). Categorical variables were presented as the number (percentage). Hepatic decompensation was defined as the presence of varices rupture, overt hepatic encephalopathy, ascites, jaundice, or an increase in Child-Pugh score of at least two points [33]. Progression-free survival, OS, time to progression to CP-B, time to ascites formation and time to hepatic decompensation were generated using Kaplan-Meier method. Patients were followed until the death or were censored at the last visit before 31 August 2019. Multivariate cox proportional hazard model was used to identify the predictive factors. Because among the explanatory variables, the correlations between ALBI grade and Child-Pugh score, between BCLC stage and PVTT, and between BCLC stage and bile duction invasion were observed and the number of the explanatory variables involved in each model depends on the number of events, authors built the three, three and four models in the analysis of OS, time to progression to CP-B and time to ascites formation in order to avoid the multicollinearity and overfitting, respectively. Platelet count was dichotomized based on the optimum cut-off value, which was determined according to the ROC curve. The factors affecting the candidate for the post-progression treatment were analyzed by using logistic regression model. Three models were built to avoid the multicollinearity and overfitting. The number of patients who fulfilled the criteria for candidate for ramucirumab treatment were so small that authors did not conduct the multivariate analysis to identify the predictive factors. Friedman test and Bonferroni method were used to compare the paired data. A value of *p* < 0.05 was considered to be statistically significant. All statistical analyses were performed using the IBM Statistical Package for the Social Sciences software version 24 (IBM SPSS 24, IBM, Armonk, NY, USA).

## 5. Conclusions

Male, ALBI grade 1, CP5A and BCLC early or intermediate stage were favorable factors related to sustaining liver function during the lenvatinib treatment and the patient with ALBI grade 1 and CP5A were eligible for the post-progression treatment. Careful screening for ascites was needed in patients with low platelet count and CP6A during lenvatinib treatment.

## Figures and Tables

**Table 1:** Patient characteristics (*n* = 110).

Age (years)	73 (66.7–80.0)
Males, *n* (%)	88 (80.0)
PS, 0 / 1 / 2, *n* (%)	77 (70.0) / 27 (24.5) / 6 (5.5)
Body weight (kg)	59.6 (53.0–66.2)
BMI (kg/m^2^)	22.8 (21.1–25.3)
Liver diseases,	
HBV / HCV / Alcohol / Others, *n* (%)	12 (10.9) / 58 (52.7) / 19 (17.3) / 21 (19.1)
Antiviral therapy for HBV, *n* (%)	8 (66.7)
HCV eradication, *n* (%)	32 (55.2)
Child-Pugh score 5 / 6, *n* (%)	58 (52.7) / 52 (47.3)
ALBI score	−2.36 (−2.70 to −2.06)
ALBI grade 1 / 2, *n* (%)	38 (34.5) / 72 (65.5)
Albumin (g/dL)	3.6 (3.4–4.1)
Total bilirubin (mg/dL)	0.73 (0.60–1.10)
Prothrombin time (%)	92 (82–101)
Platelet count (×10^4^/μL)	13.6 (9.9–19.0)
Treatment-naïve, *n* (%)	20 (18.2)
History of hepatic resection, *n* (%)	35 (31.8)
History of RFA, *n* (%)	43 (39.1)
History of TACE, *n* (%)	93 (84.5)
The number of previous TACE treatments	2 (1–6)
History of molecular targeted therapies, *n* (%)	26 (23.6)
Esophageal varices, None / F1 / F2, *n* (%) *	82 (76.6) / 16 (15.0) / 9 (8.4)
History of treatment for esophageal varices, *n* (%)	9 (8.2)
Initial dose, 12 mg / 8 mg / 4 mg, *n* (%)	22 (20.0) / 58 (52.7) / 30 (27.3)
Full dose / Reduced dose, *n* (%)	58 (52.7) / 52 (47.3)
BCLC stage, *n* (%)	
Early / Intermediate / Advanced	3 (2.7) / 33 (30.0) / 74 (67.3)
Extrahepatic spread, *n* (%)	36 (32.7)
Macroscopic vascular invasion, *n* (%)	22 (20.0)
Bile duct invasion, *n* (%)	10 (9.1)
PVTT, *n* (%)	15 (13.6)
AFP ≧ 200 ng/mL, *n* (%)	38 (34.5)
DCP ≧ 200 mAU/mL, *n* (%)	57 (51.8)

Continuous variables were expressed as the median (interquartile range). Categorical variables were expressed as the number (percentage). * Three patients did not receive esophagogastroduodenoscopy. AFP: α-fetoprotein, ALBI grade: albumin-bilirubin grade, BCLC: Barcelona Clinic Liver Cancer, BMI: body mass index, DCP: des-γ-carboxy prothrombin, HBV: hepatitis B virus, HCV: hepatitis C virus, PS: performance status, PVTT: portal vein tumor thrombus, RFA: radiofrequency ablation, TACE: transcatheter arterial chemoembolization.

**Table 2:** Assessment of tumor response (*n* = 90).

CR	1 (1.1)
PR	26 (28.9)
SD	44 (48.9)
PD	19 (21.1)
Objective response rate (%)	30.0
Disease control rate (%)	83.3

Twenty patients have not been evaluated. Data was expressed as the number (percentage). CR: complete response, PD: progressive disease, PR: partial response, SD: stable disease.

**Table 3:** Results of multivariate analyses relevant to overall survival in all patients (*n* = 110) and in patients treated with lenvatinib as first-line treatment (*n* = 84)

		Model 1		Model 2		Model 3	
Variable		Hazard Ratio (95% CI)	*p*-Value	Hazard Ratio (95% CI)	*p*-Value	Hazard Ratio (95% CI)	*p*-Value
**All patients**							
Sex	Male	1	0.24	1	0.26	1	0.17
	Female	1.62 (0.72–3.62)		1.60 (0.71–3.60)		1.77 (0.78–4.01)	
Age (years)	≧ 73	1	0.060	1	0.057	1	0.071
	< 73	1.45 (0.25–1.03)		0.50 (0.25–1.02)		0.52 (0.26–1.06)	
ALBI grade	Grade 1	1	0.059				
	Grade 2	0.45 (0.19–1.03)					
Child-Pugh score	5			1	0.46		
	6			1.30 (0.65–2.59)			
BCLC stage	Intermediate, Early					1	0.045
	Advanced					2.50 (1.02–6.10)	
**Patients treated with lenvatinib as first-line treatment**							
Age (years)	≧ 73	1	0.110	1	0.11	1	0.096
	< 73	0.48 (0.20–1.17)		0.48 (0.20–1.17)		0.47 (0.19–1.14)	
ALBI grade	Grade 1	1	0.36				
	Grade 2	1.52 (0.62–3.69)					
Child-Pugh score	5			1	0.88		
	6			1.07 (0.46–2.48)			
BCLC stage	Intermediate, Early					1	0.087
	Advanced					2.39 (0.88–6.48)	

ALBI grade: albumin-bilirubin grade, BCLC: Barcelona Clinic Liver Cancer, CI: confidence interval.

**Table 4:** Results of a multivariate analysis of factors associated with the time to progression to Child-Pugh class B (*n* = 110).

		Model 1		Model 2		Model 3	
Variable		Hazard Ratio (95% CI)	*p*-Value	Hazard Ratio (95% CI)	*p*-Value	Hazard Ratio (95% CI)	*p*-Value
Sex	Male	1	0.021	1	0.024	1	0.016
	Female	2.27 (1.12–4.54)		2.24 (1.11–4.53)		2.33 (1.17–4.65)	
Platelet count	≧12 × 10^4^/μL	1	0.24				
	<12 × 10^4^/μL	1.45 (0.78–2.70)					
ALBI grade	Grade 1	1	0.030			1	0.033
	Grade 2	2.38 (1.09–5.26)				2.35 (1.07–5.15)	
Child-Pugh score	5			1	0.037		
	6			2.03 (1.04–3.95)			
BCLC stage	Intermediate, Early	1	0.048				
	Advanced	2.08 (1.01–4.17)					
PVTT	Presence			1	0.29		
	Absence			0.64 (0.28–1.46)			
Bile duct invasion	Presence					1	0.44
	Absence					1.76 (0.42–7.32)	
Previous TACE	≧2			1	0.39		
	<1			1.32 (0.70–2.48)			
Initial dose	Full dose					1	0.53
	Reduced dose					1.22 (0.66–2.24)	

ALBI grade: albumin-bilirubin grade, BCLC: Barcelona Clinic Liver Cancer, CI: confidence interval, PVTT: portal vein tumor thrombus, TACE: transcatheter arterial chemoembolization.

**Table 5:** Results of a multivariate analysis of factors associated with ascites formation (*n* = 110).

		Model 1		Model 2		Model 3		Model 4	
Variable		Hazard Ratio (95% CI)	*p*-Value	Hazard Ratio (95% CI)	*p*-Value	Hazard Ratio (95% CI)	*p*-Value	Hazard Ratio (95% CI)	*p*-Value
Sex	Male	1	0.33	1	0.75	1	0.093	1	0.17
	Female	1.52 (0.66–3.45)		1.15 (0.49–2.70)		2.02 (0.89–4.58)		1.51 (0.67–3.45)	
Platelet count	≧ 12 × 10^4^/μL	1	0.013	1	0.010				
	< 12 × 10^4^/μL	2.56 (1.22–5.26)		2.63 (1.25–5.26)					
ALBI grade	Grade 1	1	0.59						
	Grade 2	1.25 (0.55–2.86)							
Child-Pugh score	5			1	0.041				
	6			2.17 (1.03–4.55)					
BCLC stage	Intermediate, Early					1	0.90		
	Advanced					0.96 (0.46–1.99)			
PVTT	Presence							1	0.28
	Absence							0.60 (0.24–1.52)	
Previous TACE	≧2					1	0.57		
	<1					0.81 (0.39–169)			
Initial dose	Full dose							1	0.92
	Reduced dose							1.04 (0.51–2.10)	

ALBI grade: albumin-bilirubin grade, BCLC: Barcelona Clinic Liver Cancer, CI: confidence interval, PVTT: portal vein tumor thrombus, TACE: transcatheter arterial chemoembolization.

**Table 6:** Results of a multivariate analysis of factors associated with candidacy for post-progression treatment (*n* = 79).

		Model 1		Model 2		Model 3	
Variable		Odds Ratio (95% CI)	*p*-Value	Odds Ratio (95% CI)	*p*-Value	Odds Ratio (95% CI)	*p*-Value
Sex	Male	1.70 (0.54–5.32)	0.36	1.83 (0.57–5.88)	0.31	2.04 (0.65–6.38)	0.22
	Female	1		1		1	
ALBI grade	Grade 1	3.24 (1.20–8.76)	0.020				
	Grade 2	1					
Child-Pugh score	5			3.80 (1.45–9.95)	0.007		
	6			1			
BCLC stage	Intermediate, Early	0.95 (0.35–2.62)	0.93				
	Advanced	1					
PVTT	Presence			0.92 (0.24–3.49)	0.91		
	Absence			1			
Bile duct invasion	Presence					1.41 (0.33–6.12)	0.64
	Absence					1	
Initial dose	Full dose					2.92 (1.15–7.44)	0.024
	Reduced dose					1	

ALBI grade: albumin-bilirubin grade, BCLC: Barcelona Clinic Liver Cancer, CI: confidence interval, PVTT: portal vein tumor thrombus.

**Table 7:** Relationship between the initial dose and liver function and between the initial dose and PS (*n* = 79).

	Full Dose Group (*n* = 35)	Reduced Dose Group (*n* = 44)
ALBI score	−2.41 (−2.79 to −1.97)	−2.34 (−2.66 to −2.07)
ALBI grade 1	14 (40.0)	12 (27.3)
Child-Pugh score 5	17 (48.6)	24 (54.5)
PS 0	29 (82.9)	29 (65.9)

Continuous variables were expressed as the median (interquartile range). Categorical variables were expressed as the number (percentage). ALBI: albumin-bilirubin, PS: performance status.
